# Large Vessel Vasculitis Occurring in Rheumatoid Arthritis Patient under Anti-TNF Therapy

**DOI:** 10.1155/2014/624184

**Published:** 2014-12-03

**Authors:** Valentina Cestelli, Amelia Spinella, Federica Campomori, Carmela Esposito, Sara Ciaffi, Gilda Sandri, Clodoveo Ferri

**Affiliations:** ^1^Rheumatology Unit, Department of Medical and Surgical Sciences for Children and Adults, University of Modena and Reggio Emilia, Via del Pozzo No. 71, 41124 Modena, Italy; ^2^Rheumatology Unit, Federico II University Hospital of Napoli, Via S. Pansini No. 5, 80131 Napoli, Italy; ^3^Emergency Medicine Unit, Department of Medicine, Emergency Medicine and Medical Specialities, University of Modena and Reggio Emilia, Via del Pozzo No. 71, 41124 Modena, Italy; ^4^Department of Diagnostic, Clinical and Public Health Medicine, University of Modena and Reggio Emilia, Via del Pozzo No. 71, 41124 Modena, Italy

## Abstract

Vasculitis is a heterogeneous group of disorders characterized by the presence of necrotic inflammatory phenomena and destruction of blood vessels. Vasculitis is classified as primary (idiopathic) or secondary to infections, connective tissue diseases and drugs but can also be considered as a paraneoplastic phenomenon. Evidence shows that the increasing use of biological agents results in a growing number of reports of autoimmune diseases induced by these therapies. An inflammatory articular chronic disease such as rheumatoid arthritis may be complicated by extra-articular manifestations, such as cutaneous or systemic vasculitis. Herewith, we describe the case of a great vessels arteritis in a patient affected by rheumatoid arthritis in therapy with an anti-TNF agent (etanercept).

## 1. Introduction

Inhibitors of tumor necrosis factor (TNF) are an effective therapy for rheumatic and systemic autoimmune diseases but the increasing use of these agents has caused secondary autoimmune conditions, such as vasculitis. Moreover, we know that rheumatoid arthritis (RA) is an inflammatory chronic articular disease that can be complicated by extra-articular manifestations, especially in cases of aggressive, long standing, seropositive RA. In our report, we describe the case of a great vessels arteritis in a patient with RA in therapy with etanercept.

## 2. Case Presentation

**Figure 1:**
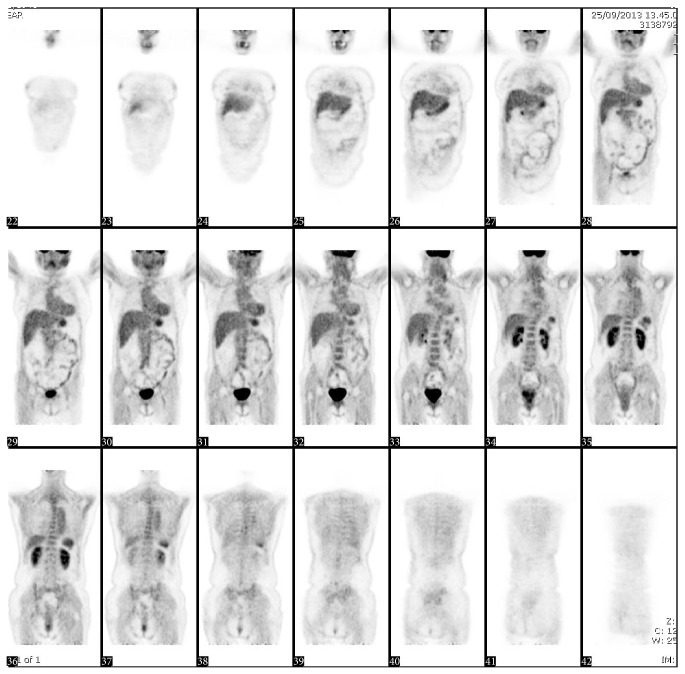
Positron emission tomography: increased uptake by the thoracic and abdominal aorta and aortic arch, indicating great vessels arteritis.

**Figure 2:**
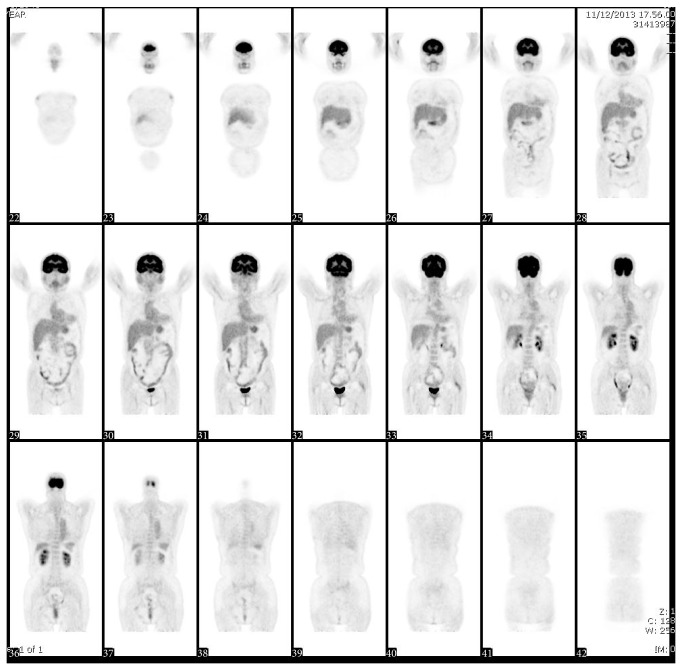
Positron emission tomography 3 months after the initiation of cyclophosphamide and steroids therapy.

A 65-year-old Italian female was formerly diagnosed with seropositive (rheumatoid factor (RF) and anticitrullinated protein antibodies (anti-CCP)) RA with etanercept treatment since 2007. The inflammatory disease was in clinical, biohumoral, and ultrasonographic remission for two years at least. The patient had previously undergone therapy with DMARDs (hydroxychloroquine, leflunomide, and methotrexate) and oral corticosteroids without significant benefits. Her medical history was otherwise relevant just for RA. She was admitted for a few days to the Emergency Room for the onset of headache and persistent abdominal pain with nausea. After admission to the Emergency Department, no signs of systemic inflammation nor any focus of infections were identified. During the hospitalization, an abdominal ultrasonography and a gastroscopy were performed to exclude acute gastritis or colelitiasis, and detected hepatic steatosis and acute gastritis with hiatal hernia. Laboratory exams showed elevated markers of inflammation (C-reactive protein (CRP) 28,35 mg/dL; D-dimer 6530 ng/mL). A thoracoabdominal angio-CT was performed suspecting arterial thrombosis and revealed a parietal thrombosis of the common hepatic artery with focal dissection, a focal thrombosis of superior mesenteric artery, an infarction of the lower pole of the right kidney and the upper third of the spleen. The autoimmunity markers as well as the cancer markers were negative. Therefore, an anticoagulant therapy was started. Subsequently, suspecting vasculitis as a feasible paraneoplastic manifestation, a positron emission tomography (PET) was necessary and revealed a flogistic involvement of the entire aorta (Figure 1). In conclusion, the diagnostic hypothesis was either RA-related-aortitis or a side effect to etanercept. The anti-TNF therapy was stopped and glucocorticoid therapy was started at doses of 1 mg/kg/daily in association with one dose of intravenous pulse cyclophosphamide (750 mg). This latter treatment was repeated after two and three months. Laboratory tests and clinical examinations showed a favorable outcome just one month later, also confirmed by a negative PET scan control performed after three months (Figure 2). The patient is still followed in our unit and continues treatment with daily oral cyclophosphamide (100 mg), daily prednisone (25 mg), and ongoing anticoagulant therapy. Markers of inflammation have normalized and the patient reported an improvement of her clinical status.

## 3. Discussion

The primary systemic vasculitides are heterogeneous, multisystem disorders characterized by inflammation and necrosis of small and medium blood vessels. Their aetiology is unknown. Vasculitic syndromes are considered in the differential diagnosis of patients with multisystem illness or pyrexia of unknown origin. However, there are a number of specific conditions that can mimic vasculitis, including infections and noninfectious inflammatory diseases, malignancy, and drugs. Vasculitis occurs commonly in the context of other autoimmune connective tissue diseases such as systemic lupus erythematosus and RA.

In our patient, infective agents, as possible triggers of a vasculitic process, have also been excluded. In literature, few cases of vasculitides due to infections are reported indeed; generally, these are forms of cutaneous vasculitis. Moreover, the manifestation of infective endocarditis often resembles vasculitis. The possibility of underlying infectious diseases in patients presenting symptoms and signs of vasculitis and vice versa should always be considered before diagnosing vasculitis [1]. Considering large vessels disease, it is known that infectious aortitis is a rare but life-threatening disorder mostly associated with abdominal aortic aneurysms; they may result from bacteremia and embolization of infectious material, which cause superinfection of a diseased and roughened atherosclerotic surface [2].

We have considered the differential diagnosis with a paraneoplastic vasculitis in our clinical case too, but this was not confirmed by laboratory and instrumental investigations. The association between cancer and vasculitis is uncommon and difficult to establish. Overall, paraneoplastic vasculitides are estimated to represent about 2–5% of all vasculitides. The only cancer-specific association with a given vasculitis is that of hairy cell leukemia and polyarteritis nodosa that typically affect medium-sized muscular arteries, also described in a variety of other malignancies. However, few cases of large-vessel vasculitis primarily occurring in the setting of myelodysplastic syndromes and myeloproliferative disorders are described in the published literature [3, 4].

As previously mentioned, we have observed the relationship between vasculitis and anti-TNF therapy in the literature. So TNF has been successfully targeted in the management of adult inflammatory arthritis to produce remission of disease and improvement in radiological progression [5]. Although these agents have been studied during the past and have demonstrated acceptable profiles of tolerability and safety, there are a growing number of reports of adverse events such as infection, an increased risk of solid tumors and lymphoma, cardiovascular disease, demyelinating disorders, and autoimmune processes ranging from asymptomatic immunological alterations to life-threatening systemic autoimmune disease. In 2006, the Study Group on Autoimmune Diseases (GEAS) of the Spanish Society of Internal Medicine created a multicenter study on the use of biological agents in adult patients with systemic autoimmune disease. This study also aimed to evaluate the occurrence of autoimmune disorders secondary to the use of biological agents, such as vasculitis, systemic lupus erythematosus/lupus-like syndrome, psoriasis, interstitial lung disease, antiphospholipid syndrome, ocular autoimmune diseases, sarcoidosis, autoimmune hepatitis, and inflammatory myopathies. In this study, 118 patients (the majority with RA) developed vasculitis after starting anti-TNF-agents. In 51% of the patients, the biological agent was etanercept, in 43% infliximab and in 4% adalimumab. Vasculitis occurred as cutaneous lesions in 86% of cases; only 2 patients presented involvement of large arteries. The mechanisms that might participate in the induction of this latter in patients receiving TNF blockers are not fully understood. It has been suggested that anti-TNF-*α*/TNF-*α* immune complexes could be deposited in small capillaries, where they activate complement and thereby trigger a type III hypersensitivity reaction [6]. The etiologic role of anti-TNF could be justified by several factors: the occurrence of vasculitis was associated with the initiation of therapy and resolved in more cases after discontinuation of biological agents; the cutaneous lesions started at the injection site (particularly in patients in therapy with etanercept) [7–9]. The genetic predisposition is an important factor to consider in these cases [10, 11].

Recently there have been reports of large vessels vasculitis (Takayasu's arteritis (TA)) in patients receiving anti-TNF-therapy [12]. In another report, Mariani et al. described the first case of TA in RA patients under anti-TNF-therapy (adalimumab) [13]. The development of a vasculitis in a patient with an inflammatory disease during biologic therapy may be due to an imbalance in immune homeostasis.

Vascular involvement is a fundamental part of the pathogenesis of RA. The vascular lesions of the blood vessels in RA are mostly asymptomatic but occasionally may cause severe manifestations. Approximately 2%–5% of RA patients develop rheumatoid vasculitis (RV), an extra-articular manifestation affecting small and medium-size arteries.

The concept of RV was first developed when vasculitis with important clinical manifestations occurred in RA patients. RV was so considered when clinical expressions of vasculitis were unexplained by other conditions such as diabetes, atherosclerosis, infections, drugs, malignancies, and other vasculitides [14]. Although the pathogenetic mechanisms underlying systemic vasculitis are still unclear, immune complexes probably play a great role. This is supported by the strong association of systemic vasculitis with higher titers of RF. Deposition of immune complexes can induce flogistic cascade with the release of cytokines, including TNF-*α*, IL-1, and IL-6. Flogistic processes of RA involve capillaries, arterioles, and venules just from the early stages, with progressive degenerative changes of the endothelium and the possible formation of thrombi, with resulting localized ischemia. The vessels most commonly involved are those of the skin and vasa nervorum of the peripheral nerves, whereas the central nervous system, eyes, heart, lungs, kidneys, and gastrointestinal system are less frequently affected [15]. The typical patient with RV has a long severe history of rheumatic disease. RA that causes aortitis is mostly a severe, seropositive, and nodular disease, associated with extra-articular vasculitic manifestations [16] and the strongest association with the development of RV is an increased titer of RF. A possible approach to the diagnosis of RV is a set of criteria proposed by Scott and Bacon in 1984 [17]. We also observed the onset of large-vessel arteritis (TA specifically) in patients with a history of RA. Although the relationship between RA and TA is unknown it can be assumed that a vascular nonspecific activation of a chronic inflammatory disorder in the presence of a predisposing genetic background can lead to the development of this disease [18, 19].

As mentioned, our patient was considered in clinical, biohumoral, and ultrasonographic remission, without extra-articular manifestations. Therefore, a strong correlation with RV is not clear indeed. In the medical literature, 213 cases of TNF-induced vasculitis have been reported: most of the patients had RA. Although the spectrum of vasculitis was broad, there was a predominance of cutaneous involvement but an important finding was the presence of systemic vasculitis in an equally large number of patients.

It can be challenging to determine causes of vasculitis associated with anti-TNF-therapy; the resolution of vasculitis after drug therapy discontinuation and adjuvant treatment is often helpful in supporting the etiologic role of anti-TNF-therapy in the development of vasculitis [9].

## 4. Conclusion

In our report, the patient developed a condition of vasculitis during treatment with anti-TNF. At the time of admission we evaluated the possible causes of her clinical manifestations. We excluded the main infectious and paraneoplastic forms, through specific instrumental and laboratory investigations. In literature, some authors have reported cases of vasculitis during treatment with biological drugs, which gradually improved after high doses of steroids and immunosuppressive therapy and the discontinuation of anti-TNF. We can also assume that the aortitis could result from the complication of the chronic, inflammatory articular disease.

Our report demonstrates the development of a great vessel vasculitis in a RA patient under treatment with etanercept. Large cohort studies of patients under biological therapy could help us to understand the relationship between vascular disease and drugs and to find patients that could develop systemic vasculitis. Moreover, all patients with inflammatory arthritis must be carefully monitored for the risk of development of an occlusive arterial involvement, with or without anti-TNF-therapy.
